# Melting, Solidification, and Viscosity Properties of Multicomponent Fe-Cu-Nb-Mo-Si-B Alloys with Low Aluminum Addition

**DOI:** 10.3390/ma17020474

**Published:** 2024-01-19

**Authors:** Yuri N. Starodubtsev, Vladimir S. Tsepelev, Viktor V. Konashkov, Nadezhda P. Tsepeleva

**Affiliations:** 1 Gammamet Research and Production Enterprise, Yekaterinburg 620131, Russia; yunstar@mail.ru; 2 Research Center for Physics of Metal Liquid, Ural Federal University, Yekaterinburg 620002, Russia; v.v.konashkov@urfu.ru (V.V.K.); n.p.konovalova@urfu.ru (N.P.T.)

**Keywords:** multicomponent alloys, melting, solidification, viscosity, liquid–liquid structure transition, clusters, activation energy

## Abstract

Melting, solidification, and viscosity properties of multicomponent Fe-Cu-Nb-Mo-Si-B alloys with low aluminum addition (up to 0.42 at.% Al) were studied using an oscillating cup viscometer. It is shown that melting and solidification are divided into two stages with a knee point at 1461 K. The temperature dependences of the liquid fraction between the liquidus and solidus temperatures during melting and solidification are calculated. It has been proven that aluminum accelerates the processes of melting and solidification and leads to an increase in liquidus and solidus temperatures. In the liquid state at temperatures above 1700 K in an alloy with a low aluminum content, the activation energy of viscous flow increases. This growth was associated with the liquid–liquid structure transition, caused by the formation of large clusters based on the metastable Fe_23_B_6_ phase. Aluminum atoms attract iron and boron atoms and contribute to the formation of clusters based on the Fe_2_AlB_2_ phase and metastable phases of a higher order.

## 1. Introduction

The multicomponent alloys contain several basic chemical elements. Each element plays an important role in achieving the declared properties of the material. In the classical soft magnetic nanocrystalline Fe_73.5_Cu_1_Nb_3_Si_13.5_B_9_ alloy, boron and silicon contribute to the formation of an amorphous structure in a thin ribbon after rapid quenching of the melt [1]. At the initial stage of heat treatment, the clustering of copper atoms occurs. Copper clusters create concentration heterogeneity and contribute to the uniform distribution of nuclei of the α-Fe–Si crystalline phase. Niobium, which is insoluble in iron, inhibits crystal growth and promotes the formation of nanocrystalline grains. Some other elements, for example, Mo, can also be inhibitors. After crystallization, a material is obtained with a crystal grain size of about 10 nm, a coercive force of less than 1 A/m, and an initial permeability of about 100,000. Iron-soluble Ni and Co are introduced to increase the efficiency of heat treatment in a magnetic field, which ensures high linearity or rectangularity of the magnetic hysteresis loop [2]. Soft magnetic nanocrystalline materials are used for transformers and electrical reactors or chokes, as well as for other electromagnetic power electronics components at frequencies up to 10 MHz [1].

Aluminum also has high solubility in iron, and the addition of aluminum leads to an increase in the initial permeability of the soft magnetic nanocrystalline material [3]. On the other hand, aluminum has high chemical activity and, interacting with oxygen, forms aluminum oxide Al_2_O_3_. Aluminum also reacts with moisture to form aluminum oxide and hydrogen. A thin film of aluminum oxide protects against oxygen penetration deep into the metal. Without a protective film, aluminum turns into loose aluminum oxide powder. Aluminum may be present in the alloy as an undesirable impurity. The high affinity of aluminum for oxygen places high demands on the oxygen and moisture content at different stages of alloy production. Aluminum reacts with nitrogen at temperatures above 1100 K to form nitride AlN.

The production of a soft magnetic nanocrystalline alloy begins with smelting. At high temperatures in the liquid state, different atoms interact with each other, forming short-range order structures, or clusters [4]. The composition, structure, and size of clusters, as well as their stability, depend on temperature, and their change is associated with the liquid–liquid structure transition (LLST) [5,6,7,8]. As a result of the LLST, the density, viscosity, electrical resistance, and other macroscopic characteristics of the melt change [7,8,9,10]. After melting the alloy, rapid quenching of the melt follows with the formation of a thin amorphous ribbon, which is a precursor for further heat treatment. The amorphous precursor inherits the short-range order structure of the metallic liquid. It is shown in [11] that the structural heredity recorded in an amorphous ribbon Fe_72.5_Cu_1_Nb_2_Mo_1.5_Si_14_B_9_ after rapid quenching of the melt remains after several heating–cooling cycles of the melt.

Among all the macroscopic properties of liquid metals, viscosity is the most sensitive to structural transformations. In a liquid nanocrystalline iron-based alloy, the Arrhenius flow predominates. In this case, the Arrhenius plot, i.e., the dependence of the natural logarithm of the kinematic viscosity lnν on the reciprocal absolute temperature *T*^−1^, is a linear function in a wide temperature range [12]. Anomalies in fluid flow manifest themselves in changes in the activation energy of viscous flow during the transition from a low-temperature region to a high temperature and vice versa. Moreover, the activation energy increases with the increasing size of clusters participating in the viscous flow of the melt [13].

Arrhenius plots during heating and cooling can form a hysteresis loop [8]. The nature of hysteresis is associated with a large number of metastable states. Therefore, the trajectory along which the melt structure will change can be very different. Also the activation energy of the viscous flow at the cooling stage and, consequently, the melt structure depends on the overheating temperature of the melt. It follows from this that, during solidification, we can obtain a material with a different structure depending on the overheating temperature of the melt. Therefore, the main task of preparing the melt is to select the optimal temperature for overheating the melt before quenching.

To determine the viscosity of metallic liquids with a high melting point, an oscillating cup viscometer is most often used [14,15,16]. In this viscometer, the sample is placed in a crucible suspended from a torsion wire that forms a pendulum. The pendulum is driven into torsional vibrations around a vertical axis. The movement of the pendulum gradually fades due to friction inside the viscous liquid. The viscosity of the liquid is calculated from the results of measuring the decrement and oscillation period. All studies using an oscillating cup viscometer refer to the temperature range in which the sample is in a liquid state. However, in multicomponent alloys, there is a significant region of coexistence of liquid and solid phases. Therefore, the purpose of this article, along with studying the effect of small aluminum additions on the kinematic viscosity of multicomponent Fe-Cu-Nb-Mo-Si-B melts, is also to evaluate the applicability of the oscillating cup viscometer, allowing for the study of the region of coexistence of liquid and solid phases during the melting and solidification phases.

## 2. Materials and Methods

Fe_72.5−x_Al_x_Cu_1_Nb_2_Mo_1.5_Si_14_B_9_ master alloys with Al = 0.02 and 0.42 at.% were smelted in an induction vacuum furnace at a temperature of 1820 K and cooled in a flat mold. Alloy samples with Al = 0.22 at.% were obtained by mixing the master alloys. The main chemical elements of the alloys were determined using a DFS-500 Optical Emission Spectrometer, OKB Spectr, Saint-Petersburg, Russia. An ARL 4460 Metals Analyzer Thermo Fisher Scientific, Walthan, MA, USA, was used to determine the O and N content. In master alloys with Al = 0.02 and 0.42 at.%, the oxygen content was 94 and 680 ppm, and the nitrogen content was 20 and 60 ppm, respectively. The error in determining the chemical composition was 1%.

The kinematic viscosity was measured by an oscillating cup viscometer in an atmosphere of pure helium. The sample was placed in a beryllium oxide cup suspended by a torsion wire. The sample was kept at a predetermined temperature for 5 min to stabilize the structural state. The kinematic viscosity was calculated from the results of measuring the decrement and period of oscillation based on the Shvidkovskiy algorithm [17]. To solve the partial differential equations for the fluid flow, we used the method of successive approximations. The error in measuring the kinematic viscosity was 3%.

The relationship between kinematic viscosity and decrement is exponential with a high adjusted coefficient of determination *R*^2^*_adj_* = 0.99998. Viscosity increases with increasing decrement; therefore, the temperature dependences of kinematic viscosity and decrement have the same form, and only the ordinate scales differ. For analysis, we will use data for both decrement and kinematic viscosity. The Shvidkovskiy algorithm can only be used for samples that are completely liquid. Therefore, on the temperature dependences, the experimental points for liquid and liquid–solid states are marked with different colors.

## 3. Results and Discussion

**Figure 1:**
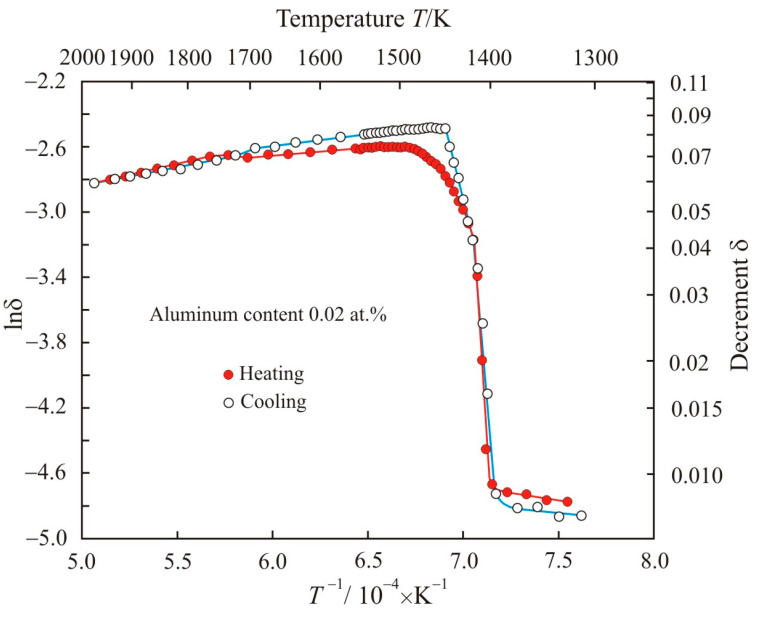
Dependences of natural logarithm of decrement lnδ on the reciprocal temperature *T*^−1^ when the Fe_72.5−x_Al_x_Cu_1_Nb_2_Mo_1.5_Si_14_B_9_ alloy with aluminum content 0.02 at.% is heated to a temperature of 1975 K and cooled.

Figure 1 shows dependences of natural logarithm of decrement lnδ on the reciprocal temperature *T*^−1^ when the Fe_72.5−x_Al_x_Cu_1_Nb_2_Mo_1.5_Si_14_B_9_ alloy with aluminum content 0.02 at.% is heated to a temperature of 1975 K and cooled. In the low-temperature region, the decrement remains almost unchanged at the level of 0.008. When heated above 1400 K, the decrement sharply increases to δ = 0.04, and, in this temperature range, the relationship between lnδ and *T*^−1^ is linear. Furthermore, the decrement increases nonlinearly, reaching a maximum value at a temperature of about 1500 K. Starting from this temperature, the decrement, as well as the kinematic viscosity of the melt, decreases. A decrease in viscosity with temperature is a typical manifestation of the liquid state of a substance. Therefore, the temperature at which the decrement reaches its maximum value will be taken as the liquidus temperature of the multicomponent alloy *T_liq_* = 1523 K. This value is close to the liquidus temperature of 1520 K of the ternary Fe_80_Si_10_B_10_ alloy [18], in which the content of basic elements is comparable to our Fe_72.5_Cu_1_Nb_2_Mo_1.5_Si_14_B_9_ alloy. On the other hand, in the low-temperature region, the temperature at which the decrement almost does not change will be taken as the solidus temperature *T_sol_* = 1393 K. Then, a sharp change in the decrement between these temperatures will correspond to the region of coexistence of the liquid and solid phases.

From Figure 1, it follows that, at the cooling stage, the liquidus temperature of the melt with aluminum content 0.02 at.% decreased to 1448 K; however, the solidus temperature did not change. Noteworthy is the linear dependence of the logarithm of decrement lnδ on the reciprocal temperature *T*^−1^ at the initial stage of solidification, in contrast to the exponential dependence at the final stage of melting. Both dependences during heating and cooling intersect at the point with coordinates lnδ = −3.2 (δ = 0.04) and *T* = 1416 K. With a further decrease in temperature, the experimental points at the stages of heating and cooling lie on the same straight line:

(1) $$\ln\delta =90.03-\frac{14.18\cdot 10^{4}}{T}$$

with an adjusted coefficient of determination *R*^2^*_adj_* = 0.991, and the decrement is related to temperature by an exponential function:

(2) $$\delta =1.38\cdot 10^{42}\exp\left(\frac{-14.18\cdot 10^{4}}{T}\right)$$

**Figure 2:**
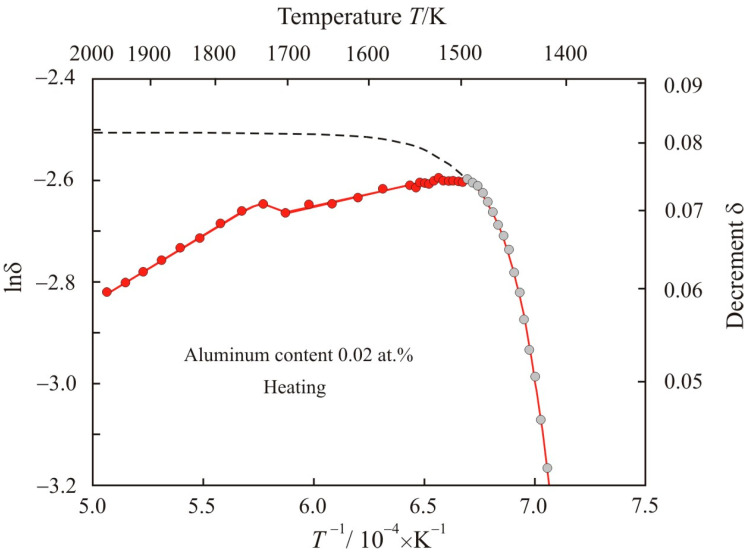
Dependence of natural logarithm of decrement lnδ on the reciprocal temperature *T*^−1^ when Fe_72.5−x_Al_x_Cu_1_Nb_2_Mo_1.5_Si_14_B_9_ alloy with aluminum content 0.02 at.% is heated to a temperature of 1975 K. The dashed line corresponds to the exponential function obtained by fitting the experimental gray points. Solid–liquid state is highlighted in gray.

Figure 2 shows the dependence of natural logarithm of decrement lnδ on the reciprocal temperature *T*^−1^ when the Fe_72.5−x_Al_x_Cu_1_Nb_2_Mo_1.5_Si_14_B_9_ alloy with aluminum content 0.02 at.% is heated to a temperature of 1975 K. Figure 2 is limited to the range of values lnδ = −3.2. Here at the initial stage of melting, the fitting of the experimental gray points corresponds to the exponential function:

(3) $$\ln\delta =-2.51-3.44\cdot 10^{-19}\exp\left(\frac{5.97\cdot 10^{4}}{T}\right)$$

with an adjusted coefficient of determination *R*^2^*_adj_* = 0.998. As temperature increases, the exponential function (3) quickly approaches saturation lnδ = −2.51; this is shown by the dashed line in Figure 2.

**Figure 3:**
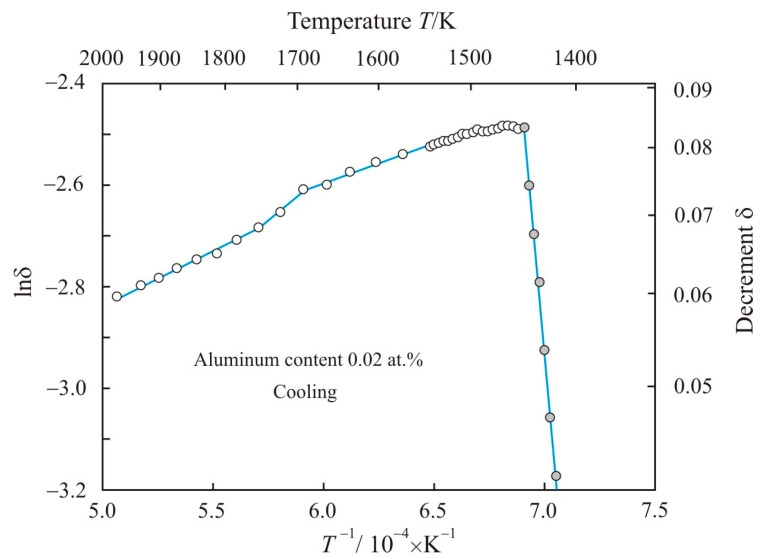
Dependence of natural logarithm of decrement lnδ on the reciprocal temperature *T*^−1^ when Fe_72.5−x_Al_x_Cu_1_Nb_2_Mo_1.5_Si_14_B_9_ alloy with aluminum content 0.02 at.% is cooled from a temperature of 1975 K. Solid–liquid state is highlighted in gray.

Figure 3 shows the dependence of natural logarithm of decrement lnδ on the reciprocal temperature *T*^−1^ when the Fe_72.5−x_Al_x_Cu_1_Nb_2_Mo_1.5_Si_14_B_9_ alloy with aluminum content 0.02 at.% is cooled from a temperature of 1975 K. During cooling, the liquidus temperature decreased by 55 K to 1448 K. At the initial stage of solidification, the fitting of the experimental gray points corresponds to the linear function:

(4) $$\ln\delta =-31.8-\frac{4.97\cdot 10^{4}}{T}$$

with an adjusted coefficient of determination *R*^2^*_adj_* = 0.997, so the decrement is related to the temperature exponential function:

(5) $$\delta =6.74\cdot 10^{13}\exp\left(\frac{-4.97\cdot 10^{4}}{T}\right)$$

**Figure 4:**
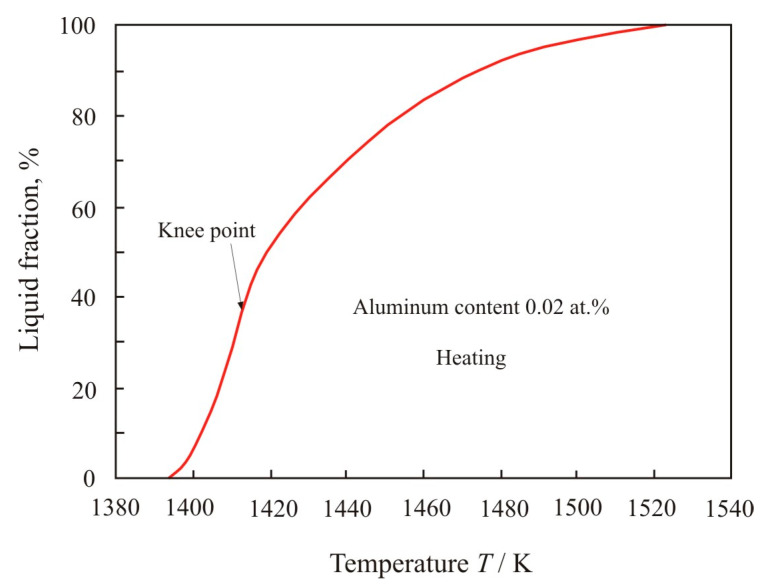
Dependence of liquid fraction on the temperature *T* when the Fe_72.5−x_Al_x_Cu_1_Nb_2_Mo_1.5_Si_14_B_9_ alloy with aluminum content 0.02 at.% is heated in the range from *T_sol_* = 1393 K to *T_liq_* = 1523 K.

In a multicomponent alloy between the solidus and liquidus temperatures, there are solid and liquid phases. The decrement is proportional to the volume of the liquid phase, in which the movement of the pendulum is damped due to friction inside the viscous liquid. Using the temperature dependences of the decrement (1) and (3), it is possible to calculate the fraction of the liquid phase depending on the temperature. Figure 4 shows the dependence of the liquid fraction on temperature when the Fe_72.5−x_Al_x_Cu_1_Nb_2_Mo_1.5_Si_14_B_9_ alloy with aluminum content 0.02 at.% heated within the temperature range from *T_sol_* = 1393 K to *T_liq_* = 1523 K. In knee point, which corresponds to a temperature of 1416 K, the functional dependence of the decrement changes during the heating process. Up to the knee point, the volume of the liquid phase grows rapidly, and then, with increasing temperature, the growth slows down.

The soft magnetic nanocrystalline Fe_73.5_Cu_1_Nb_3_Si_13.5_B_9_ alloy has a chemical composition close to eutectic and consists of crystalline grains about 10 nm in size, which are separated by an amorphous matrix. The crystalline phase accounts for about 70% of the volume [1]. Nanocrystals have a composition close to Fe_80_Si_20_, and they contain elements soluble in iron. Insoluble elements such as boron and niobium remain at the periphery in the amorphous matrix. Melting begins along the grain boundaries [19] with the formation of a liquid phase of eutectic composition. It can be assumed that above the knee point, stable phases melt, mainly Fe_2_Si, Fe_2_Si_0.4_B_0.6,_ and Fe_2_B [18,20]. A variety of stable phases slows down the melting process at temperatures higher the knee point in Figure 4.

**Figure 5:**
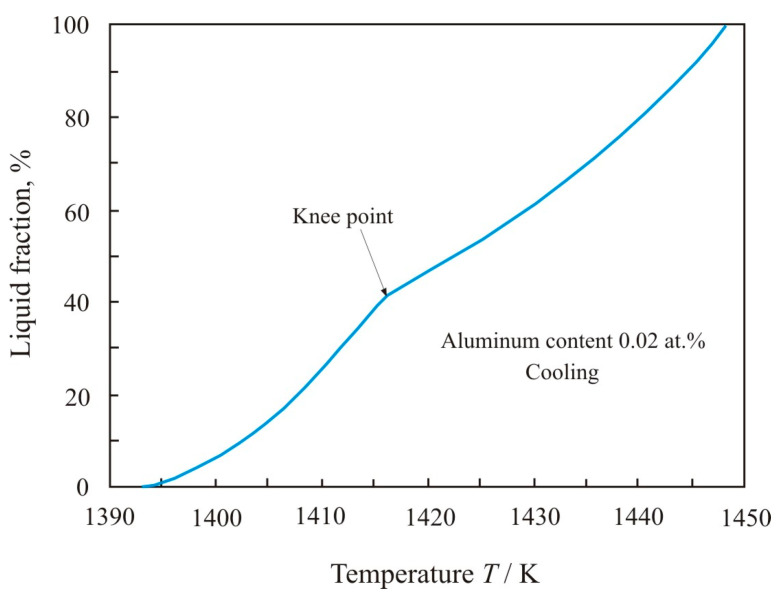
Dependence of liquid fraction on the temperature *T* when the Fe_72.5−x_Al_x_Cu_1_Nb_2_Mo_1.5_Si_14_B_9_ alloy with aluminum content 0.02 at.% is cooled in the range from *T_liq_* = 1448 K to *T_sol_* = 1393 K.

Cooling of the Fe_72.5−x_Al_x_Cu_1_Nb_2_Mo_1.5_Si_14_B_9_ melt with aluminum content 0.02 at.% begins at a high temperature of 1975 K. At this temperature, the atoms are weakly bonded to each other. The melt is characterized by a large number of metastable states. Therefore, melt solidification pathways with temperature can vary greatly. When cooling from a high temperature, the metastable phases Fe_3_B and Fe_23_B_6_ [21] have an advantage, and they effectively suppress the formation of stable phases and lead to the undercooling of the melt. Figure 5 shows the dependence of the liquid fraction on the temperature *T* when the Fe_72.5−x_Al_x_Cu_1_Nb_2_Mo_1.5_Si_14_B_9_ alloy with aluminum content 0.02 at.% is cooled in the range from *T_liq_* = 1448 K to *T_sol_* = 1393 K. The form of the obtained dependences is typical for alloys with a wide region of coexistence of liquid and solid phases [22,23], and knee points on these curves coincide with the crystallization of stable phases. From Figure 5, it follows that, upon cooling, the volume of the liquid phase decreases more significantly at the final stage when the eutectic phase solidifies. The knee points during melting and solidification coincide, since the same phases of the multicomponent alloy participate in the processes.

Table 1 shows melting and solidification parameters in the range from liquidus temperature to knee point 1416 K for Fe_72.5−x_Al_x_Cu_1_Nb_2_Mo_1.5_Si_14_B_9_ alloys with aluminum content 0.02; 0.22 and 0.42 at.%. Coefficients *A*_0_ and *B* correspond to the approximation of experimental values in the form of an exponential function at the final stage of melting, starting from the knee point 1416 K:

(6) $$\ln\delta =A_{0}+A_{1}\exp\left(\frac{B\cdot 10^{4}}{T}\right)$$

and coefficient *b* corresponds to the approximation of experimental values in the form of a linear function at the initial stage of solidification up to knee point 1416 K:

(7) $$\ln\delta =a+\frac{b\cdot 10^{4}}{T}$$

Table 1 shows that, with increasing aluminum content, the liquidus and solidus temperatures, as well as the coefficients *B* and *b*, increase. An increase in the coefficients indicates that the temperature dependences are becoming steeper. Consequently, small additions of aluminum accelerate the melting and solidification processes. This acceleration of processes is quite expected since aluminum actively binds with other elements. Such a stable compound may be Fe_2_AlB_2_, the formation energy of which, according to various sources [24,25,26], is higher than the formation energy of the most stable Fe_2_B boride. Table 2 shows design parameters of the compounds Fe-B. An additional Fe_2_AlB_2_ phase with high formation energy can increase the solidus and liquidus temperatures.

The temperature dependence of kinematic viscosity can be represented as an Arrhenius-type equation:

(8) $$\nu =\nu_{0}e^{\frac{E_{a}}{RT}}$$

where ν is the kinematic viscosity (m^2^·s^−1^), ν_0_ is a pre-exponential factor with the dimension of the kinematic viscosity, *E_a_* is the activation energy of the viscous flow (J·mol^−1^), *R* is the gas constant (J·K^−1^·mol^−1^), and *T* is the absolute temperature (K). Taking the logarithm of Equation (1), we obtain Arrhenius plot:

(9) $$\ln\nu =\ln\nu_{0}+\frac{E_{a}}{RT}$$

where *E_a_*·(*RT*)^−1^ is the reduced activation energy, which compares the activation energy *E_a_* with thermal energy *RT*. At constants of *E_a_* and ν_0_, the logarithm of the kinematic viscosity is a linear function of the inverse absolute temperature.

**Figure 6:**
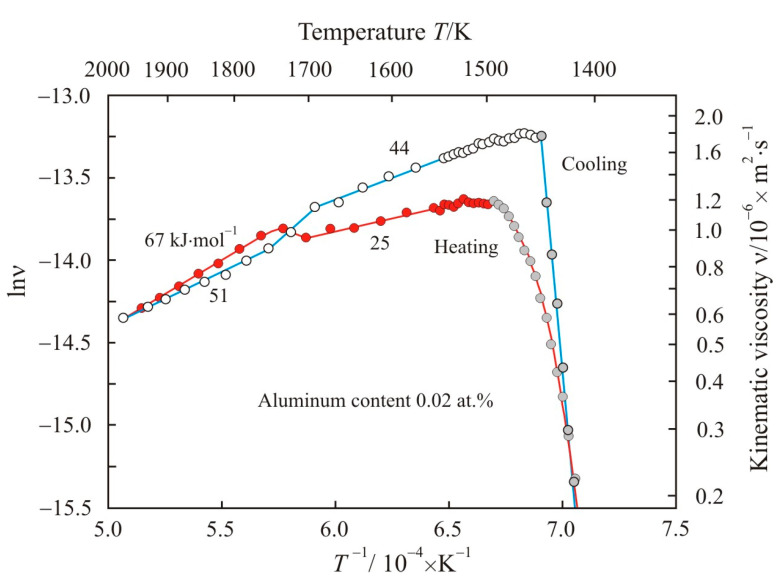
Dependences of natural logarithm of kinematic viscosity lnν on the reciprocal temperature *T*^−1^ when the Fe_72.5−x_Al_x_Cu_1_Nb_2_Mo_1.5_Si_14_B_9_ alloy with aluminum content 0.02 at.% is heated to a temperature of 1975 K and cooled. Solid–liquid state is highlighted in gray. The numbers next to the curves show the activation energy of the viscous flow *E_a_*.

Figure 6 shows Arrhenius plots as the dependences of the natural logarithm of the kinematic viscosity lnν on the reciprocal temperature *T*^−1^ when the Fe_72.5−x_Al_x_Cu_1_Nb_2_Mo_1.5_Si_14_B_9_ alloy with aluminum content 0.02 at.% is heated to a temperature of 1975 K and cooled. The numbers next to the curves show the activation energy of the viscous flow *E_a_* in the corresponding linear sections of the Arrhenius plots. It follows from Figure 6 that, when heated in a low-temperature region, the activation energy has a low value of 25 kJ·mol^−1^. At a temperature of about 1700 K, the Arrhenius plot switches to a new trajectory with a high activation energy of 67 kJ·mol^−1^. The cooling trajectory of the melt from a temperature of 1975 K does not coincide with the heating trajectory. During cooling, the transition to a new trajectory also occurs at a temperature of about 1700 K, and the activation energy in the low-temperature region is equal 44 kJ·mol^−1^. Thus, in the low-temperature region, the activation energy at the cooling stage is higher than during heating.

Previously [8], it was shown that, at a constant temperature, the activation energy is higher when larger clusters participate in the viscous flow of a liquid. An increase in activation energy with increasing cluster size is typical, not only for viscous fluid flow, but for other transport phenomena [28,29,30]. Thus, when heated in a low-temperature region with the lowest activation energy, small clusters contribute to the viscous flow of the melt. For example, in a Co-B melt, an activation energy of 25 kJ·mol^−1^ corresponds to clusters with a size of about 0.15 nm, i.e., on the order of atomic size [8]. Figure 6 shows that immediately after melting at a temperature of about 1500 K, the slope of the Arrhenius plot corresponds to an activation energy of even less than 25 kJ·mol^−1^, and it is comparable to the thermal energy *RT* = 12.5 kJ·mol^−1^ at this temperature. High thermal energy increases the mobility of atoms, which freely overcome the potential barrier *E_a_*. As temperature increases, the number of free atoms increases. Free atoms can move over significant distances and, interacting with each other, form new clusters.

Electronegativity on the Luo–Benson scale for Fe and B are 1.77 and 3.66, respectively [31]. Boron atoms are most strongly associated with iron atoms since this pair of elements has the distinct electronegativity. Table 2 shows design parameters of the compounds Fe-B. Stable Fe_2_B boride with high formation energy predominates in the structure of the Fe_72.5_Cu_1_Nb_2_Mo_1.5_Si_14_B_9_ alloy at the initial stage of melting. Therefore, when heated in a low-temperature region, the melt contains Fe_2_B-based clusters [18,20], along with free atoms of other elements. In the Fe_72.5_Cu_1_Nb_2_Mo_1.5_Si_14_B_9_ alloy, the ratio of iron atoms to boron atoms is 89:11. This ratio is much greater than that for the Fe_2_B compound in which iron and boron atoms are bonded. As temperature increases, the mobility of atoms increases and the bonds between atoms weaken. But, at the same time, the probability of free iron atoms approaching boron increases. In a cluster based on Fe_3_B, the ratio of bound iron atoms to boron atoms is 3:1. In a cluster based on Fe_23_B_6_, the ratio is 79:21, and it is much closer to Fe_72.5_Cu_1_Nb_2_Mo_1.5_Si_14_B_9_. Thus, in the cluster structure based on Fe_23_B_6_, boron atoms bind almost all iron atoms in the Fe_72.5_Cu_1_Nb_2_Mo_1.5_Si_14_B_9_ melt, thereby ensuring a more uniform distribution of atoms in the melt at high temperatures.

The direction of clustering from clusters based on Fe_2_B to large clusters based on Fe_23_B_6_ is a consequence of the tendency of a multicomponent melt to a homogeneous structure at high temperature. This LLST occurs at a temperature of about 1700 K and is manifested in Figure 6 as an increase in the activation energy of viscous flow. Note that in [32], using differential scanning calorimetry, an endothermic peak was discovered at 1687 K in liquid Fe_78_Si_9_B_13_, which confirms the structural transition in the melt at this temperature.

At the cooling stage, at a temperature of about 1700 K, a reverse structural transition occurs with a decrease in the activation energy to 44 kJ·mol^−1^ (see Figure 6). A slight decrease in the activation energy indicates that, in this temperature region, clusters based on Fe_23_B_6_ are retained in the melt. The preservation of such clusters confirms the discovery of the metastable Fe_23_B_6_ phase in undercooled eutectic Fe_83_B_17_ alloys in the solid state [21].

In the eutectic melt Co_81.4_B_18.6_, LLST was also recorded with a transition to a short-range order of Co_23_B_6_ with a high activation energy of about 70 kJ·mol^−1^ [8]. A decrease in melt density was also detected in this region. This confirms the presence of clusters based on Co_23_B_6_, which have the largest molar volume. However, in the high-temperature region, the activation energy in the Co_81.4_B_18.6_ melt drops again to 19 kJ·mol^−1^, and this drop indicates the predominance of atomic-sized clusters in the melt. In the Fe_72.5−x_Al_x_Cu_1_Nb_2_Mo_1.5_Si_14_B_9_ alloy with aluminum content 0.02 at.%, the temperature range with high activation energy is maintained up to a maximum temperature of 1975 K. This can be explained by the greater stability of iron borides compared to cobalt borides. This follows from a comparison of the formation energy of the compounds Co_23_B_6_, Co_3_B, and Co_2_B, which is −0.089; −0.133; and −0.175 eV·atom^−1^ [8], respectively, with data for iron borides in Table 2.

**Figure 7:**
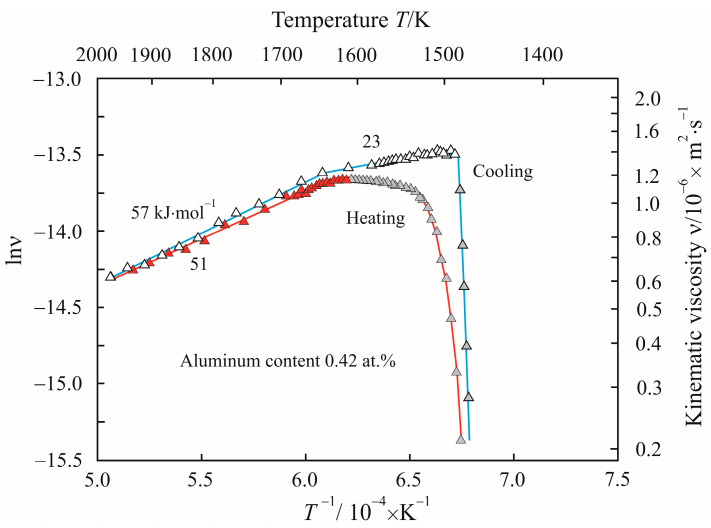
Dependences of natural logarithm of kinematic viscosity lnν on the reciprocal temperature *T*^−1^ when the Fe_72.5−x_Al_x_Cu_1_Nb_2_Mo_1.5_Si_14_B_9_ alloy with aluminum content 0.42 at.% is heated to a temperature of 1975 K and cooled. Solid–liquid state is highlighted in gray. The numbers next to the curves show the activation energy of the viscous flow *E_a_*.

Figure 7 shows Arrhenius plots as the dependences of the natural logarithm of the kinematic viscosity lnν on the reciprocal temperature *T*^−1^ when the Fe_72.5−x_Al_x_Cu_1_Nb_2_Mo_1.5_Si_14_B_9_ alloy with aluminum content 0.42 at.% is heated to a temperature of 1975 K and cooled. The numbers next to the curves show the activation energy of the viscous flow *E_a_* in the corresponding linear sections of the Arrhenius plots. From a comparison of Figure 6 and Figure 7, it follows that the addition of aluminum almost did not change the viscosity of the melt at a maximum temperature of 1975 K, but the trajectory of the Arrhenius plots noticeably changed. Figure 7 shows that a transition to high activation energy is observed above 1650 K during heating and cooling. The constancy of the activation energy indicates an insignificant change in the structure of the melt over a wide temperature range. The electronegativity of aluminum on the Luo–Benson scale is 2.40 and is midway between iron and boron. Therefore, aluminum atoms attract both iron atoms and boron atoms with the formation of clusters, based on the stable ternary phase Fe_2_AlB_2_ [29,30] with high formation energy (see Table 2) or higher-order metastable phases Fe_3_AlB_4_ and Fe_4_AlB_6_ [33].

## 4. Conclusions

Using an oscillating cup viscometer, the melting, solidification, and viscous properties of a multicomponent Fe_72.5−x_Al_x_Cu_1_Nb_2_Mo_1.5_Si_14_B_9_ alloy with aluminum content up to 0.42 at.% Al were studied. The decrement is proportional to the volume of the liquid phase, in which the movement of the pendulum is damped due to friction inside the viscous liquid. This made it possible to use an oscillating cup viscometer to study liquid and solid–liquid states. It is shown that melting and solidification are divided into two stages with a knee point at 1461 K. The temperature dependences of the liquid fraction in the alloy between the liquidus temperature and the solidus temperature during melting and solidification are calculated. The volume of the liquid phase grows faster at the initial stage of melting and at the final stage of solidification. Aluminum has been proven to accelerate the melting and solidification processes. The addition of aluminum leads to an increase in the liquidus and solidus temperatures.

In the liquid state, at a temperature of about 1700 K in an alloy with a low aluminum content, a liquid-liquid structure transition was detected, which manifests itself in a change in the activation energy of viscous flow. The activation energy is higher in the high-temperature region, in which large clusters based on the metastable Fe_23_B_6_ phase are formed as a result of a liquid–liquid structural transition. When cooled, the cluster structure of Fe_23_B_6_ contributes to a decrease in the liquidus temperature. Aluminum atoms attract iron and boron atoms and contribute to the formation of clusters based on the stable Fe_2_AlB_2_ phase and metastable phases of a higher order. A melt with a high aluminum content has a high activation energy at temperatures above 1650 K.

## Figures and Tables

**Table 1:** Melting and solidification parameters in the range from liquidus temperature to knee point 1416 K for Fe_72.5−x_Al_x_Cu_1_Nb_2_Mo_1.5_Si_14_B_9_ alloys with aluminum content 0.02; 0.22 and 0.42 at.%.

Al, at.%	Heating			Cooling		
	*T_liq_*, K	*A*_0_	*B*, K	*T_liq_*, K	*T_sol_*, K	*b*, K
0.02	1448	−2.51	5.97	1523	1393	−4.97
0.22	1463	−2.76	9.20	1559	1427	−7.68
0.42	1472	−2.80	14.91	1619	1457	−15.35

**Table 2:** Design parameters of the compounds Fe-B.

Compound	Formation Energy/eV·atom^−1^ [27]	Mole Volume/10^−6^ × m^3^·mole^−1^
Fe_23_B_6_ Fe_3_B Fe_2_B Fe_2_AlB_2_	−0.169 −0.213 −0.307 −0.538 [24] −0.366 [26]	6.288 5.914 5.503 5.576

## Data Availability

Data are contained within the article.
